# Polyurethanes Modified by Ionic Liquids and Their Applications

**DOI:** 10.3390/ijms241411627

**Published:** 2023-07-19

**Authors:** Xue Wang, Zhenjie Zhao, Meiyu Zhang, Yongri Liang, Yingdan Liu

**Affiliations:** State Key Laboratory of Metastable Materials Science and Technology, College of Materials Science and Engineering, Yanshan University, Qinhuangdao 066004, China; wx13303287141@163.com (X.W.); zhenjie_shine@163.com (Z.Z.); zhangmeiyu_1998@163.com (M.Z.)

**Keywords:** polyurethane, ionic liquids, blending, copolymerization, application

## Abstract

Polyurethane (PU) refers to the polymer containing carbamate groups in its molecular structure, generally obtained by the reaction of isocyanate and alcohol. Because of its flexible formulation, diverse product forms, and excellent performance, it has been widely used in mechanical engineering, electronic equipment, biomedical applications, etc. Through physical or chemical methods, ionic groups are introduced into PU, which gives PU electrical conductivity, flame-retardant, and antistatic properties, thus expanding the application fields of PU, especially in flexible devices such as sensors, actuators, and functional membranes for batteries and gas absorption. In this review, we firstly introduced the characteristics of PU in chemical and microphase structures and their related physical and chemical performance. To improve the performance of PU, ionic liquids (ILs) were applied in the processing or synthesis of PU, resulting in a new type of PU called ionic PU. In the following part of this review, we mainly summarized the fabrication methods of IL-modified PUs via physical blending and the chemical copolymerization method. Then, we summarized the research progress of the applications for IL-modified PUs in different fields, including sensors, actuators, transistors, antistatic films, etc. Finally, we discussed the future development trends and challenges faced by IL-modified PUs.

## 1. Introduction

**Figure 1:**
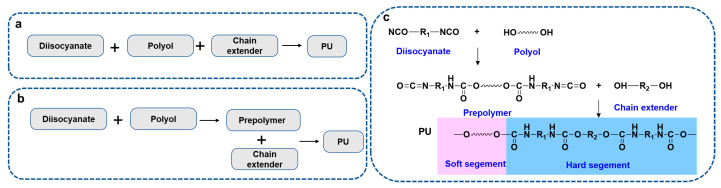
Synthesis route of PU by a one-step method (**a**) and a prepolymer method (**b**) and the chemical structures of the reactants, prepolymer and PU, with soft and hard segments (**c**).

Polyurethane (PU) is a kind of polymer with carbamate groups in its chain structure, which is generally prepared by the reaction of isocyanate, polyol, and chain extenders via a one-step or prepolymer method [1,2]. PU has attracted much attention due to its high strength, good wear resistance, excellent structural designability, and processability. It has been widely used in fields such as mechanical engineering [3,4], electronic equipment [5,6], and medical treatment [7,8]. PU is a block polymer containing repeated carbamate segments in the main molecular chain, among which the segments with a glass transition temperature above room temperature are called rigid or hard segments, and those with a glass transition temperature below room temperature are called flexible or soft segments [9,10]. The hard segment is composed of polyisocyanates and chain extenders, in which polyisocyanates can be divided into aromatic and aliphatic and the chain extenders are small molecular polyols or polyamines [11,12]. The soft segment is polyols, and according to their molecular structure, the PUs can be divided into the types of polyester, polyether, polycarbonate, and polyolefin [13]. The schematic synthesis process of PU by a one-step method and a prepolymer method, and the basic chemical structures of PU are shown in Figure 1.

The mechanical performance and application of PUs greatly depend on the chemical structures of soft/hard segments and chain extenders, the cross-linking density, and the interaction between the hard and soft segments [14,15]. The type of soft and hard segments affects the hardness, strength and toughness, and thermal stability of PUs. In addition, hydrogen bonding, crystallinity, cross-linking density, and molecular weight are all important factors affecting the performance of PU. As a physical cross-linking point, hydrogen bonds give PU high mechanical strength and toughness, and the dynamic association–dissociation mechanism of hydrogen bonds also helps to restore mechanical properties [16]. Proper cross-linking can improve the strength and toughness of PU elastomers [17], but excessive cross-linking will lead to a decrease in mechanical properties and make the PU brittle. To a certain extent, an increase in the molecular weight will improve the tensile strength, elongation at break, and wear resistance [18]. In addition to the above-mentioned factors, the physical properties of PUs in a wide range can be explained by the two-phase morphology, which is formed due to the partial incompatibility of soft and hard segments. The hard segment of PUs generally contains strong polar groups of urethane, the large cohesion energy of which leads to the formation of hydrogen bonds and hard microdomains by further aggregation [19]. By contrast, soft microdomains are composed by the weak polar polyether or polyester segments [20]. There is a part of miscibility between the hard and soft segments; however, the hard and soft microdomains are thermodynamically incompatible [21] due to the existence of a large number of hydrogen bonds between the hard segment. Therefore, the hard microdomains cannot be completely dissolved in the soft phase, but they exist in the soft phase as a physical cross-linking point, showing the microphase separation structure [22,23,24]. This unique microphase separation structure endows PUs with good mechanical properties, where the hard segment can provide sufficient strength, while the soft segment can provide good flexibility and toughness [25]. The selection of small molecular chain extenders also has an important impact on the performance of PU. For example, Gao et al. studied the effects of different chain extenders on the degree of microphase separation and mechanical properties of PU, and demonstrated that chain extenders with amide bonds in the structure can form more ordered hydrogen bonds, resulting in high microphase separation and good mechanical properties [26]. When 4-aminophenyl disulfide is used as a chain extender for PU, it can undergo rapid and dynamic reform after the disulfide bond breaks, giving PU excellent self-healing performance [27].

In addition, adding different compounds to PU can improve its performance in certain aspects. Adding plasticizers (for example, dibutyl phthalate, triphenyl phosphate [28], dibutyl adipate [29,30], gelatin glycerin [31], and polyethylene glycol [28,32]) can effectively reduce the modulus of PU and improve its dielectric constant and polarizability. Mixing PU with nanofillers, such as graphene [33] and carbon nanotubes [34,35], can enhance the electrical and thermal conductivity of PU. The introduction of ionic liquids (ILs) can enhance the conductivity [36] and flame retardancy of PU [37]. Blending PU with other polymers (such as polyaniline [38,39] and SEBS [40]) can also acquire hybrid polymers with diverse characteristics. In summary, the performance of PU can be adjusted not only by the structures of monomers but also by blending with additives or polymers to meet specific needs of applications.

Among the various additives for PU, ILs [41] are a type of green and environmentally friendly solvent with a low melting point, incombustibility, chemical and physical stability, high ionic conductivity, and a strong interaction with polymers. Because of their excellent physical and chemical properties, ILs have been widely used in material synthesis [42,43,44] and modification and molding processing [45,46,47]. In terms of polymer modification, the application of ILs has received increasing attention. The introduction of ILs into PU, poly(vinyl chloride) (PVC) [48,49], poly(vinylidene fluoride) (PVDF) [50,51,52], polyacrylates [53,54,55], polyimide [56,57], and other polymer networks [58,59,60,61,62,63] through simple physical mixing can endow the polymers with excellent properties. For example, a trace amount of IL added to PVC can play a plasticizing role, thus reducing the modulus of PVC. It also improves the interface polarization and ion polarization abilities of PVC and enhances the electric field response of PVC-based dielectric gel [64,65]. If the content of IL in the polymer matrix is increased, ion–dipole interactions and dipole–dipole interactions will give the material high conductivity. The addition of large amounts of IL can also weaken the interaction between the polymers, giving the polymer excellent flexibility [66]. This provides a new idea for the development of flexible sensors.

IL-modified PU, which is also called ionic PU (i-PU), can be obtained by introducing ILs with various structures or specific functions into PU by physical blending or chemical copolymerization. The obtained ionic PU combines the excellent characteristics of both ILs and PU, enabling PU to possess specific properties such as conductivity [67], antistatic ability [68], flame retardancy [69], and antibacterial ability [70]. It greatly broadens the application field of PU. In this review, we will summarize the recent research progress on the ionic PU, especially the IL-modified PU, mainly including the experimental methods to synthesize ionic PUs and their performance and related applications. We will also discuss the future development trends and challenges faced by ionic PU.

## 2. Fabrication Methods of Ionic Liquid-Modified PU (PU-IL)

### 2.1. Physical Blending of ILs with PU by Solvent Casting

Physical blending is a general way to prepare polymer blends or introduce additives into the polymer matrix. To prepare IL-modified PU (PU-IL) by physical blending, solvent casting process is usually applied, which involves dissolving PU (in the form of particles or films) in organic solvent (such as N, N-dimethylformamide, or tetrahydrofuran) first, then adding a certain amount of IL into it to obtain a uniform solution through mechanical stirring, and finally volatilizing the organic solvent [36,71]. ILs exist in PU matrix in the form of anions and cations. There is electrostatic attraction between anions and cations, and there is also interaction between IL and PU matrix. This is because there are a large number of carbamate polar groups in the PU chain, which are prone to forming interactions with polar IL molecules [72]. The interaction between IL and PU destroys the interaction between hard–hard segments and hard–soft segments and disturbs the entanglement of PU polymer chains. As a result, the glass transition temperature (*T*_g_) of PU decreases, and the mobility of polymer chains is enhanced. From this point of view, IL plays a plasticizing role in PU [73]. Compared with other small molecular plasticizers, IL has a stronger interaction with PU, which can reduce the surface migration and leakage of IL molecules. If a certain amount of IL is added to PU, it not only plasticizes the PU matrix, but also endows PU with adjustable electrical properties. According to the interaction force, volume size, and content of the anions and cations in IL, the obtained PU-IL hybrid materials can be applied in various fields such as electric actuators [74], capacitance/resistance sensors [75], polymer solid-state electrolytes [76], and electrodes.

**Figure 2:**
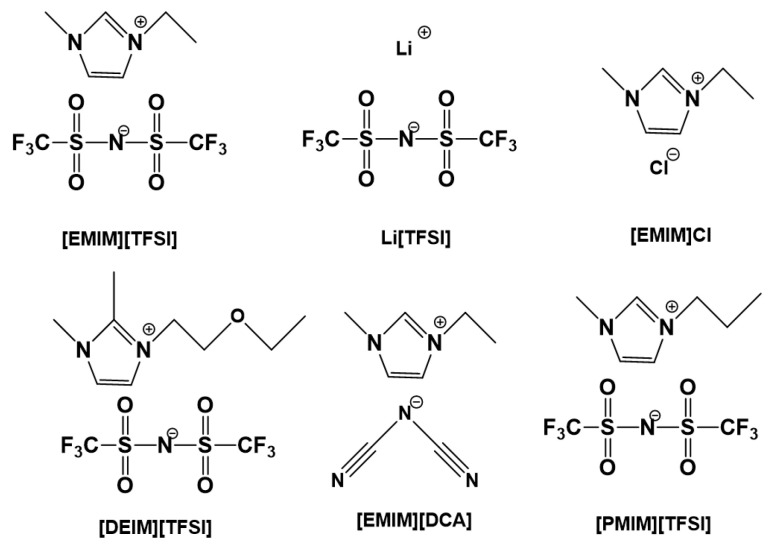
Chemical structures of ILs used for mixing with PU to prepare PU-based ionogels.

Generally, there are many kinds of ILs that can be used to modify PU by physical blending. For example, 1-ethyl-3-methylimidazolium bis(trifluoromethylsulfonyl)imide ([EMIM][TFSI]) [77,78] is usually applied to blend with PU. Its anionic group [TFSI]^−^ can easily form hydrogen bond with the carbamate group of PU. This is because [TFSI]^−^ contlyains strong electronegative elements such as N, O, and F. Jiang et al. [36] prepared high transparent and flexible PU-based ionogels by mixing PU with ILs containing [TFSI]^−^ as anions rather than [BF_4_]^−^ or [PF_6_]^−^. This indicated that the compatibility of ILs with PU was dominated by the anion rather than the cation group. The hydrogen interaction between [TFSI]^−^ and PU was confirmed by Fourier transform infrared spectroscopy. Jin et al. [73] calculated the binding energy between IL and PU using density functional theory. The results showed that the average binding energy of [TFSI]^−^ to different binding sites of hard and soft segments of PU was higher than [EMIM]^+^, which not only indicated that [EMIM][TFSI] existed in the hard and soft segments of PU, but also the interaction between anions and PU was greater than cations. Lee et al. [79] fabricated cross-linked PU-based ionogels with response ability to temperature by synthesizing PU in the presence of ILs. They found that the ionogel could undergo reversible transparent–opaque transition by heating or cooling it. In addition to [EMIM][TFSI], commonly used ILs for mixing with PU also include Li[TFSI] [77,80], 1,2-dimethyl-3-ethoxyethyl-imidazolium bis(trifluoromethanesulfonyl)imide ([DEIM][TFSI]) [81], [EMIM]Cl [82], 1-ethyl-3-methylimidazolium dicyanamide ([EMIM][DCA]) [27], and [PMIM][TFSI] [83]. The chemical structures of the applied ILs are shown in Figure 2.

### 2.2. Copolymerization of ILs with Monomers or Prepolymers for PU

In addition to physical mixing, ILs can be introduced into the polymer chain of PU by copolymerization. For physical blending, it is a facile method to prepare PU-based ionogels or elastomers; however, the weak physical interaction between IL and PU cannot avoid the problems of IL leakage and poor stability, which seriously limits the long-term application of the ionogel. The above problems can be solved by introducing IL to PU matrix by chemical reaction including copolymerization, chemical grafting, or other strong interactions to bind IL to the PU chain segment. Immobilizing ILs with special properties such as high conductivity, flame retardancy, and antibacterial properties onto the PU chains can optimize their performance and achieve multifunctional i-PU. Due to the fact that the main raw materials for synthesizing PU include isocyanates, oligomer alcohols, and small molecule chain extenders, IL-contained chemicals can be introduced into the raw materials or used directly as raw materials. To introduce ionic groups into the soft segment of PU is relatively complex; therefore, current research on the synthesis of i-PU mainly focuses on the ionic modification of hard segment. If the polymer chain of PU has C=C groups, C=C-contained ILs can react with the PU polymer chain by radical polymerization [84]. When ILs have -OH or -NH_2_ groups, they can react with isocyanate groups by step-growth polymerization. ILs with a single -OH or -NH_2_ group can be used as a capping agent for PU. For example, John et al. [85] used 1-methyl-3-hydroxyundecyl-imidazolium bromide (HOC_11_C_1_ImBr) as a capping agent and synthesized transparent PU-IL gel. The transparency of the PU-IL gel can change in different solvents, showing excellent recyclability to solvent stimulation. ILs with two or more -OH or -NH_2_ groups can be used as chain extenders [86] or cross-linkers [87,88] of PU.

**Figure 3:**
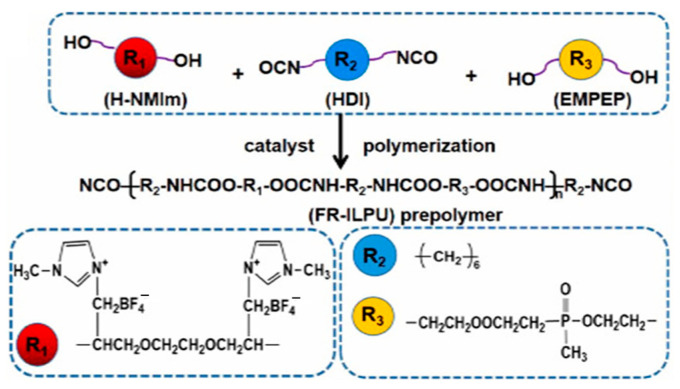
The schematic process of synthesizing flame-retardant ionic liquid polyurethane (FR-ILPU) [91]. “Reprinted/adapted with permission from Ref. [91]. 2023, Chen, J.; Rong, L.; Liu, X.; Liu, J.; Yang, X.; Jiang, X.” More details on “Copyright and Licensing” are available via the following link: https://doi.org/10.1016/j.polymer.2023.125759.

Chemically modified PU by ILs endows PU with improved mechanical and multifunctional properties. PU prepared using an ionic cross-linking agent (tris(2-hydroxyethyl) methylammonium methylsulfate) showed higher tensile strength and elongation at break due to the enhanced phase separation structure. In addition, the ionic cross-linking also improved the oil resistance of PU [89]. After applying IL with a quaternary ammonium cation as a comonomer to synthesize PU, the obtained i-PU film had antimicrobial activity against Staphylococcus aureus and Escherichia coli bacteria [90]. A new flame-retardant PU film was prepared by simultaneously grafting IL (N-methylimidazole tetrafluoroborate diethylene glycol ether, H-NMIm) and phosphorus-containing diol (ethylene glycol methyl phosphonate ethylene glycol propionate, EMPEP) into the PU segments by the reaction with hexamethylene diisocyanate (HDI). The flame-retardant IL-modified PU (FR-ILPU) film not only had excellent electrochemical performance, but also had good flame retardancy and cycle stability [91], providing a new strategy for the application of PU film in safe and flexible lithium batteries (Figure 3). A novel cationic PU was obtained using imidazole diol-based IL as a comonomer. It had better microphase separation to achieve controllable mechanical, thermal, and electrical properties [92]. When PU was used as a separation membrane, methoxysilane-functionalized IL was chemically anchored to the PU polymer chain by sol–gel reaction between methoxysilane groups of IL and the end-capped groups of PU prepolymer. The functionalized IL in PU polymer can significantly improve the selectivity of CO_2_/CH_4_ [93].

According to the charge type of ILs on the PU chain segment, i-PUs can be divided into three different types: anionic [94], cationic [95], and zwitterionic PU [96]. Saalah et al. [94] successfully introduced -COO^−^ into the PU segments by using jatropha oil, isophorone diisocyanate (IPDI), and 2, 2-bis(hydroxymethyl)propionic acid (DMPA) to synthesize an anionic waterborne PU. Cationic PU can be synthesized by polyaddition of diisocyanates with an ionic diol having a 1, 2, 3-triazolium in the backbone and a [TFSI]^−^ counter anion [97]. Using ionic cross-linker, such as tris(2-hydroxyethyl)methylammonium methylsulfate [88], is also an alternative strategy to achieve cationic PU. A PU with zwitterion moiety was synthesized by polymerization of IPDI and N-methyldiethanolamine (MDEA), and then ring-opening addition and quaternization were carried out by adding 1,3-propanesulfonic acid lactone [98].

Compared to the physical blending method, i-PU obtained by chemical copolymerization has ionic groups fixed on the PU chain segments, resulting in a more uniform distribution of ionic groups and being less prone to ion aggregation. It also has better stability, but in terms of electrical performance, the conductivity of physically obtained i-PU by double ion migration is generally higher than that of chemical copolymerization single ion migration. Sometimes, i-PU/IL was also prepared by blending chemically modified PU with ILs to achieve excellent performance, including mechanical properties, conductivity, and self-healing capabilities.

## 3. Applications of PU-IL

PU-IL obtained by modifying PU with IL with different structures and properties can meet the requirements of different applications. The ionic conductivity, ionic diffusion coefficient, viscosity, volume size, and even shape of anions and cations, as well as their interaction with polymer matrix of IL, can all affect the performance of the hybrid material of PU-IL. Due to the high conductivity and high dissociation of IL, IL molecules in PU-IL can undergo ionic polarization or migration in the soft and hard segments of PU. This specific property makes PU-IL have attractive application potential as resistive or capacitive sensors [36,99], soft actuators [77], transistors [86], and polymeric solid electrolytes [46]. The presence of conductive IL in PU matrix also enhances the antistatic ability of PU [100]. ILs containing elements such as phosphorus and boron play a key role in improving the flame retardancy of PU. This is because the IL molecules can migrate to the surface of PU, promoting the formation of a dense carbon layer on the surface of PU [101]. In addition, CO_2_ has high solubility in IL; however, pure IL has high viscosity and high cost. The polymer-IL thin film not only reduces the high-cost problem in CO_2_ absorption, but also improves the absorption capacity and rate [102,103]. Some ILs have excellent biocompatibility and antibacterial properties, which means that the PU-IL materials can be applied in the biomedical field in vivo or in vitro [104,105]. The applications of PU-IL are described in detail as follows.

### 3.1. Sensors

Sensors are a type of intelligent substance that can perceive external information such as strain, pressure [106,107], temperature [108], humidity [109], light [110], etc., and convert this information into other visible signals. In recent years, due to the development of flexible materials, flexible sensors [111] with excellent flexibility, stretchability, compressibility, bendability, and foldability have been able to replace rigid sensors that are not easily deformed to meet the requirements of wearability and lightweight. Flexible sensors can deform the attached skin or human body, and their resistance or capacitance will change with the deformation strain or pressure. Ionogels or ionic elastomers prepared by introducing IL into polymer matrix combine the ionic conductivity of IL and the flexibility of polymer matrix. The research into ionogels and ionic elastomers as flexible sensors has received widespread attention from researchers. As one of the members, i-PUs obtained by modifying PU with IL show more possibilities in structural design and make it possible to prepare multifunctional skin-like sensors to be used for monitoring of human health and environmental changes [112].

**Figure 4:**
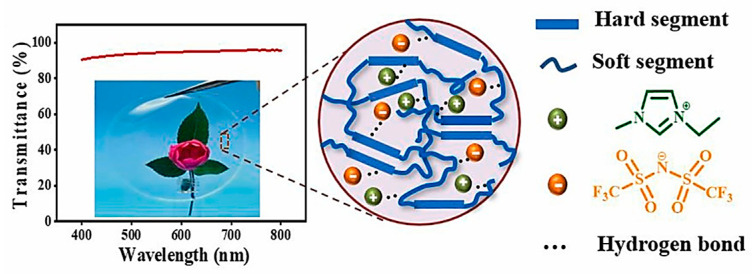
Transmittance of TPU@IL ionogel in the visible range (400–800 nm). The inset is a photo of TPU@IL ionogel, and the background of a flower can be seen clearly. The right image shows the schematic structure of the ionogel [36]. “Reprinted/adapted with permission from Ref. [36]. 2021, Jiang, N.; Chang, X.; Hu, D.; Chen, L.; Wang, Y.; Chen, J.; Zhu, Y.” More details on “Copyright and Licensing” are available via the following link: https://doi.org/10.1016/j.cej.2021.130418.

Jiang et al. [36] fabricated a skin-like sensor by mixing IL and thermoplastic polyurethane (TPU) by physical blending (Figure 4). The prepared TPU@IL ionogel as a stretchable transparent strain sensor showed a large strain sensing range of 0.1–500% and high transparency up to 94.3%. The ionogel also exhibited temperature-dependent conductivity and can be applied as a temperature sensor. Due to its low freezing point, it had a wide working temperature range from −40 to 100 °C and a high detection accuracy of 0.1 °C. At the same time, the sensor showed obvious antimicrobial ability and cytocompatibility.

**Figure 5:**
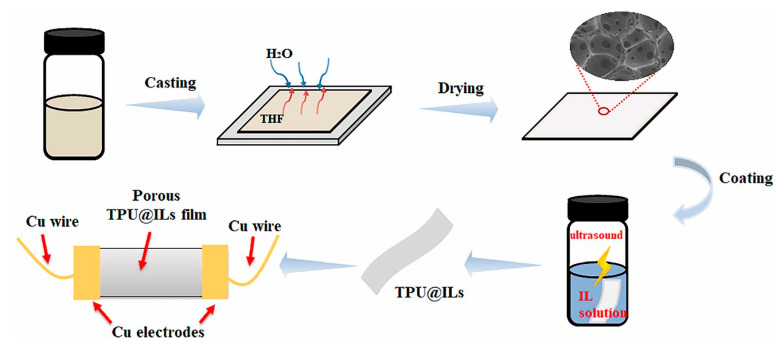
Schematic process of the fabrication of hierarchically porous TPU@ILs ionogel film and the assembly of flexible sensor based on the film [113]. “Reprinted/adapted with permission from Ref. [113]. 2022, Peng, M.; Li, X.; Liu, Y.; Chen, J.; Chang, X.; Zhu, Y.” More details on “Copyright and Licensing” are available via the following link: https://doi.org/10.1016/j.apsusc.2022.155516.

In addition to strain and temperature sensing, it is also possible to prepare solid electrolyte thin films by mixing TPU and IL ([EMIM][TFSI]) (i-TPU) as an ionic chemiresistor skin (ICS). The prepared i-TPU electrolyte showed ultra-stable and highly sensitive sensing of five organic chemical volatiles (VOC) including toluene, ethanol, propionate, acetone, and hexane. It can work well under harsh environmental conditions (relative humidity up to 85% or temperature up to 100 °C) and severe mechanical deformation (strain up to 100% or bending radius of 1 mm) [73]. This provides an idea for disease diagnosis and environmental monitoring. Using a similar preparation method, Xu et al. [78] proposed a design of interlocking hexagonal microcolumn array structures to simulate the shape of interlocking microstructures constructed on the surface of the human body. Porous TPU films can also be prepared by gas-induced phase separation [113]. Then, TPU@IL ionogel film was fabricated by immersing the porous film in IL with ultrasonication and IL diffused into the TPU matrix under the action of ultrasound. The prepared film was assembled using Cu electrodes and wires to make a flexible multi-sensory sensor (Figure 5).

Some researchers coated IL on the surface of inorganic nanostructures and then mixed the IL-modified nanomaterials with PU to prepare electronic skin sensor [99]. The anionic [TFSI]^−^ in the IL of [EMIM][TFSI] was attached to the surface of silica microspheres via hydrogen bonds with silanol groups. The cationic [EMIM]^+^ was also coated on the surface by coulomb force and π-π stacking with anions. A supramolecular bond interaction was formed between the IL-modified silica and the TPU matrix. The pressure-sensing ability was realized through the evolution of ion pairs on the surface of TPU. This ionic skin exhibited ultra-high sensitivity of 48.1–5.77 kPa^−1^ over a wide pressure range (0–135 kPa) at an ultra-low voltage of 1 mV, which surpassed pressure-sensing capabilities of various natural skin mechanoreceptors. Chen et al. [114] prepared a PU-based ionic skin for underwater epidermal biopotential monitoring by introducing fluorine-rich segment in the PU backbone that was mixed with fluorine-rich IL of [EMIM][TFSI]. The ionic conductivity of the PU elastomer can remain stable even after vigorous washing in water, which provides a new approach for sensing in underwater environments.

Chen et al. [95] synthesized a new type of ionic chain extender containing ammonium cationic groups and prepared a series of i-PU as ionic skin. The special structure of i-PU improved the interaction between PU chain and IL and limited the leakage of IL in the dielectric layer. Using a similar conception, Xu et al. [83] also mixed IL (1-propyl-3-methyl-imidazolium bis(trifluoromethyl-sulfonyl) imide, [PMIM][TFSI]) with the PU containing copolymerized ionic segments, which was synthesized using a dihydroxyl IL ([DHP-VIM][TFSI]) as a monomer. The dipole–dipole interactions and ion–dipole interactions between the two ILs enhanced the interaction between IL and the PU matrix, which improved the conductivity and stability of the i-PU. In addition, dynamic borate ester bonds were also introduced into the PU chains through the click reaction of the double bonds grafted on the dihydroxy IL monomers and the sulfhydryl group on the boronic ester, which was used as a cross-linker. It endowed the i-PU with self-healing characteristics.

### 3.2. Actuators

As a typical electro-actuator, dielectric elastomers (DEs) [115] have a wide range of applications in sensors, actuators, robots, and biomedicine due to their high energy conversion efficiency, large deformation ability, simple structure, and lightweight nature. Typical DE materials include silicone rubber (PDMS) [116], acrylic elastomers [117], PU, and block copolymers (such as styrene-butadiene-styrene and styrene-ethylene-butylene-styrene). Due to the high Young’s modulus and insufficient dielectric properties [28,29,30], PU has small electrodeformation and small driving stress, which limits the development and application of PU elastomers as flexible actuators. It is found that using IL as a low molecular plasticizer [28,31,38] for PU can not only reduce the modulus of PU but also increase the dielectric constant of PU by improving the ionic polarizability.

**Figure 6:**
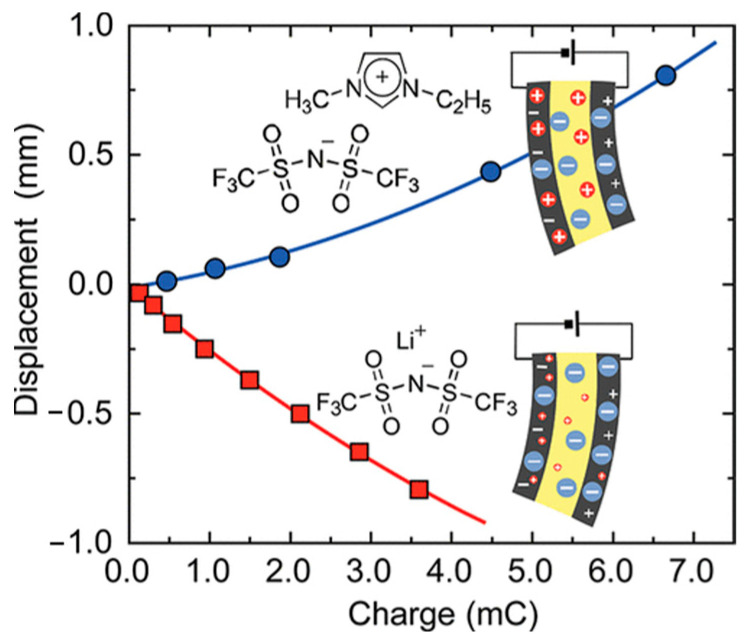
Electro-deformation mechanism of the PU/Li[TFSI] and PU/[EMIM][TFSI] actuators [77]. “Reprinted/adapted with permission from Ref. [77]. 2012, Imaizumi, S.; Kato, Y.; Kokubo, H.; Watanabe, M.” More details on “Copyright and Licensing” are available via the following link: https://doi.org/10.1021/jp301501c.

Imaizumi et al. [77] used Li[TFSI] and [EMIM][TFSI] to selectively modify the polyether segments of PU to prepare PU-based electrolytes. The ionic conductivity of PU/Li[TFSI] and PU/[EMIM][TFSI] electrolytes reached 1.4 × 10^−6^ and 3.4 × 10^−3^ S cm^−1^, respectively. The researchers discussed the difference in ion transport mechanisms between PU/Li[TFSI] and PU/[EMIM][TFSI] electrolytes from the aspects of ionic conductivity and self-diffusion coefficient and proposed a deformation model for the actuator with a solid-state electric double-layer capacitor (EDLC) structure, which is shown in Figure 6. The model indicated that the direction of deformation depends on *t*_+_*v*_+_ − *t*_−_*v*_−_ (in which *t*_+_ is the cationic transference number, *v*_+_ is the cationic volume, *t*_−_ is the anionic transference number, and *v*_−_ is the anionic volume). The deformation behavior of the two actuators of PU/Li[TFSI] and PU/[EMIM][TFSI] electrolytes was compared and was consistent with the explanation of the model.

### 3.3. Transistors

**Figure 7:**
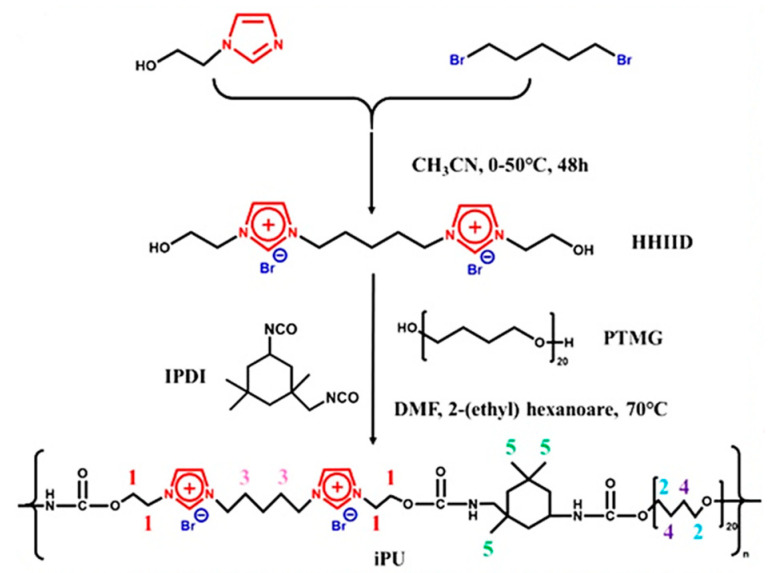
Synthesis process of ionic PU (i-PU) by IPDI, PTMG, and ionic chain extender, HHIID [86]. “Reprinted/adapted with permission from Ref. [86]. 2022, Han, S.; Zhang, R.; Han, L.; Zhao, C.; Yan, X.; Dai, M.” More details on “Copyright and Licensing” are available via the following link: https://doi.org/10.1016/j.eurpolymj.2022.111292.

Transistors are indispensable electronic components in almost all electronic products. At present, many researchers are engaged in the design and application of flexible transistors to meet the requirements of wearable devices and applications in complex conditions [118,119]. Polymer gels or elastomers containing ionic groups [120,121] have high conductivity supported by ILs and also overcome the low stability of the transistor caused by the flow of ILs. Tabi et al. [122] prepared an i-PU ionogel by dissolving the PU chip and [EMIM][TFSI] in N, N-dimethylformamide (DMF) and demonstrated a high-capacitance PU ionogel/PU bilayer gate dielectric for low-voltage and high-performance pressure-sensitive organic thin-film transistors, indicating that the charge carrier mobility increased to 2 cm^2^ V^−1^ s^−1^, and the on/off ratio reached 10^5^ at a drive voltage of 6 V. Han at el. [86] first synthesized an ionic chain extender, HHIID, and then synthesized an i-PU with poly(tetramethylene ether glycol) (PTMG), isophorone diisocyanate (IPDI), and HHIID (as shown in Figure 7). The i-PU/IL film was then prepared by mixing the i-PU with [EMIM][TFSI], showing great potential as transparent, stable, durable, and stretchable channel layers for transistors.

### 3.4. Antistatic Films

To avoid electrostatic damage caused by contact between some precision instruments and large equipment and the electric spark, the electrical resistivity of the electric spark must be reduced. The antistatic agent is generally distributed in the material or on the surface of the material, the role of which is to increase the conductivity of the material. If the material’s surface is slightly conductive, it can avoid the accumulation of charges on the surface of the material. Surface and volume resistivity, half-time of charge decay [60], and triboelectric properties are often used to evaluate the anti-electrostatic ability of the materials. In general, surface resistivity at the level of 10^10^ Ω sq^−1^ [123] is considered an excellent antistatic material. Introducing conductive fillers, such as carbon nanotubes [124], graphene [125], and IL [126,127], is considered to be an effective way to prepare antistatic materials.

Wu et al. [68] synthesized a new type of polyol by esterification of poly(1, 4-butanediol adipic glycol) (PBA) and methyl 3-(3-[2-hydroxyethyl]imidazole-1-yl) propionate chloride. Then, the IL-embedded polyol was applied to synthesize PU, which made the PU permanently antistatic because it contained IL in its backbone. The PU showed excellent mechanical and thermal properties, with elongation at break reaching 800%, breaking strength up to 14 MPa, and decomposition temperature above 230 °C. Compared with the traditional antistatic polymer by physical mixing, it avoids the issues of antistatic ability of the polymer gradually deteriorating over time, high costs, and the migration of conductive fillers.

**Figure 8:**
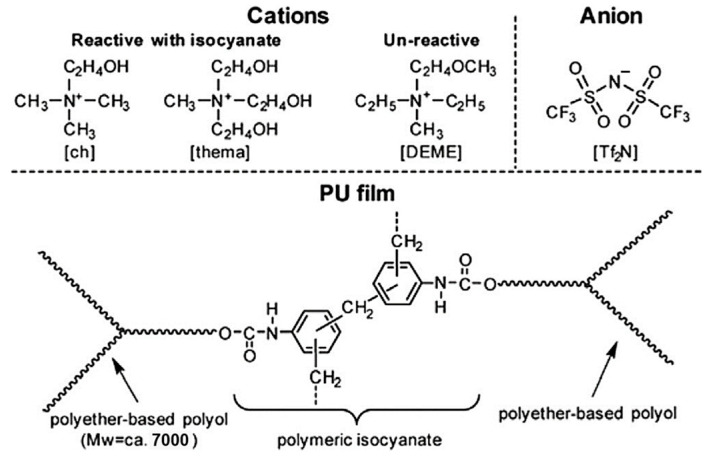
Structure of [Tf_2_N]-type ILs containing [ch] or [thema] as reactive cations and N, N-diethyl-N-methyl-N-(2-methoxyethyl)ammonium as an un-reactive cation, and the common structure of the PU films synthesized by the reaction of polymeric isocyanate and polyether-based polyol [100]. “Reprinted/adapted with permission from Ref. [100]. 2014, Iwata, T.; Tsurumaki, A.; Tajima, S.; Ohno, H.” More details on “Copyright and Licensing” are available via the following link: https://doi.org/10.1016/j.polymer.2014.03.028.

In order to explore the effect of IL species on antistatic properties, Iwata et al. [100] synthesized active ILs with terminal hydroxyl groups to prepare IL-fixed PU films. As shown in Figure 8, the bis(trifluoromethanesulfonyl)imide ([Tf_2_N])-type ILs containing (2-hydroxyethyl)trimethylammonium ([ch]) or tris(2-hydroxyethyl)methylammonium ([thema]) as cations are reactive with isocyanate. Pure PU film was prepared by the reaction of polymeric diphenylmethylene diisocyanate with trifunctional polyether polyols based on propylene-ethylene oxides (P(PO/EO)). The IL-fixed PU film was prepared by a similar process using P(PO/EO)-IL mixture instead of pure P(PO/EO) as polyol. They found that the PU film containing [Tf_2_N] counterion showed the lowest surface resistivity and permanent antistatic characteristics because of the strong covalent fixing of IL on the PU chain segment. A core–shell structured antistatic agent for PU was prepared by coating poly(ionic liquid) on the surface of poly(methyl methacrylate). The resistivity of the PU film was successfully reduced, even though the conductive fillers were solid [124]. This indicated that both IL and polymerized IL are attractive materials for antistatic polymers.

### 3.5. Solid-State Polymer Electrolytes

Solid-state electrolyte (SSE) [128,129,130] is a solid-state ionic conductive electrolyte that plays a role in separating and conducting ions in electrochemical batteries to generate current. Compared with traditional organic liquid electrolytes, SSE has various advantages, such as high ionic conductivity, good thermal stability, and electrochemical stability. Therefore, the safety of SSE-based batteries has been greatly improved. The solid-state polymer electrolyte (SPE) is an important member of SSE, which has excellent flexibility, good interface compatibility, and is lightweight. SPE can undergo varying degrees of elastic and plastic deformation to comply with changes in electrodes [131]. The polymer matrix in SPE can provide a supporting framework and transmission medium for ion transport. Generally, polymers with a high dielectric constant, polarized groups, and a low glass transition temperature are selected, such as poly(ethylene oxide) (PEO), polyacrylonitrile (PAN), poly(vinylidene fluoride) (PVDF), and their copolymers, polyamide (PA) and PU [132,133]. Introducing IL into the polymer matrix as an excellent plasticizer can improve the conductivity of SPE by providing free migrating ions through the high ionic conductivity of IL. PU has a unique microphase separation structure with polyether or polyester polyols, as its soft segments can transport Li^+^, while the hard segment composed of isocyanates and chain extenders can provide high strength. The application of ionic PU as a polymer electrolyte has attracted much attention in lithium batteries.

**Figure 9:**
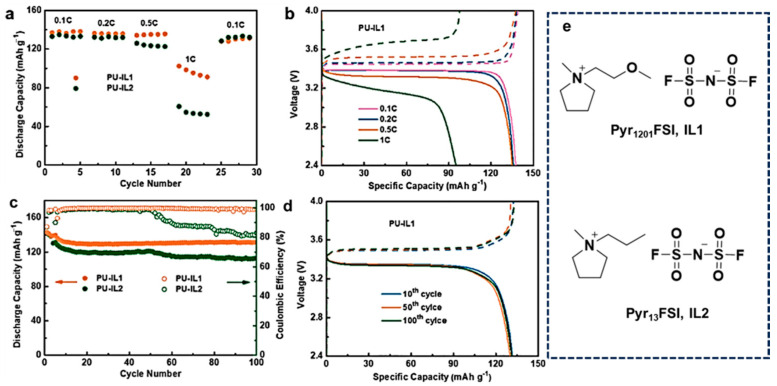
Performance of LiFePO_4_/Li batteries with PU-IL1 and PU-IL2 electrolytes at 80 °C: (**a**) rate performance of the batteries from 0.1 to 1 C; (**b**) charge–discharge profiles of the battery with PU-IL1 electrolyte at different current densities. Charge is represented by a dotted line and discharge is represented by a solid line); (**c**) cycling performance of the batteries at 0.5C; (**d**) charge–discharge curves at the 10th, 50th, and 100th cycles. Charge is represented by a dotted line and discharge is represented by a solid line) (**e**) the structure diagram of Pyr_1201_FSI (IL1) and Pyr_13_FSI (IL2) [134]. “Reprinted/adapted with permission from Ref. [134]. 2022, Fang, L.; Sun, W.; Hou, W.; Wang, Z.; Sun, K.” More details on “Copyright and Licensing” are available via the following link: https://doi.org/10.1016/j.electacta.2022.141316.

In previous studies, it has been found that at the same concentration of lithium salts, the different anionic structures of lithium salts (LiCl, LiClO_4_, and Li[TFSI]) can significantly affect the conductivity of PU-based SPE, mainly due to the interaction between charge carriers and PU. Due to the easier dissociation of [TFSI]^−^ with Li^+^ and the easy formation of hydrogen bonds with PU, PU/Li[TFSI] has a higher conductivity. Lavall et al. [46] used PU as a polymer matrix and prepared an SPE by mixing PU with an IL of PYRA_1201_[TFSI] and a lithium salt of Li[TFSI] by a casting technique, and propylene carbonate was also added to the mixture to enhance the conductivity of the SPE. Based on this technique, an advanced method was proposed by Fang et al. [134]. They prepared a heat-resistant and non-flammable PU-based electrolyte by introducing a functionalized IL N-methyl-N-methoxyethyl-pyrrolidinium bis(fluorosulfonyl)imide (Pyr_1201_FSI, IL1) and a standard IL N-methyl-N-propyl-pyrrolidinium bis (fluorosulfonyl)imide (Pyr13FSI, IL2) into a porous PU. As shown in Figure 9a,b, compared with the LiFePO_4_/Li battery with a PU-IL2 electrolyte, the battery with the PU-IL1 electrolyte showed similar discharge capacities at lower rates of 0.1 and 0.2C, but better capacities at higher rates of 0.5 and 1C. In the cycling charge–discharge profiles (Figure 9c,d), the battery showed a steady discharge capacity of 131 mA h g^−1^ and a coulombic efficiency of 99% after 100 cycles. The superior electrochemical performance of the PU-IL1 electrolyte is attributed to the ether group in IL1, which increases the flexibility of the IL, enhances the movement of ions, improves the compatibility between the IL and PU, and finally improves the conductivity of SPE.

### 3.6. Flame-Retardant Elastomers

**Figure 10:**
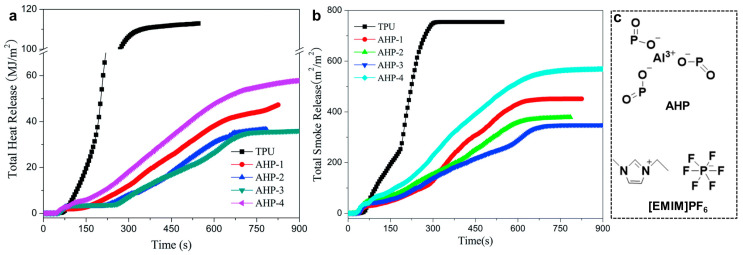
Total heat release (**a**) and total smoke release (**b**) of TPU composites at a flux of 35 kW m^−2^, and the chemical structure of AHP and [EMIM]PF_6_ (**c**). The samples AHP-1, AHP-2, AHP-3, and AHP-4 have different contents of [EMIM]PF_6_: 0 wt%, 0.03125 wt%, 0.0625 wt%, and 0.125 wt%, respectively [101]. “Reprinted/adapted with permission from Ref. [101]. 2016, Chen, X.; Ma, C.; Jiao, C.” More details on “Copyright and Licensing” are available via the following link: https://doi.org/10.1039/C6RA14094G.

PU is easy to burn when exposed to fire and will release a large number of NO, CO, and other toxic gases [135,136]; therefore, improving the flame-retardant performance of PU is of great significance. Pyrrolinium-based IL has been reported to be used as a flame retardant in lithium-ion battery electrolytes [137]. It has been found that ionic PU has better flame-retardant performance and smoke-suppression properties [138]. Imidazolium-based IL ([EMIM]PF_6_) can be used as a catalyst and auxiliary flame retardant, and aluminum hypophosphite (AHP) was used as the main flame retardant in PU by a melting blending method. As shown in Figure 10a, the total heat release values and the slope of the curves significantly decrease with the addition of AHP and [Emim]PF_6_, indicating that there is an obvious improvement in flame retardancy in the TPU composites. In Figure 10b, the total smoke release curves of flame-retardant TPU composites are much lower than those of TPU. This implies that the IL can catalyze the decomposition of AHP to form a protective carbon layer, reduce the smoke emission parameters, improve the flame-retardant effect, and further enhance the thermal stability of TPU/AHP systems [101].

In PU-based composites, IL and inorganic nanofiller have a synergistic effect on the flame-retardant properties of PU. Czlonka et al. [139] filled PU with different weight ratios of melamine, silica, and IL ([EMIM]Cl). Jiao et al. [140] prepared a PU composite using IL (1-((ethoxycarbonyl)methyl)-3-methylimidazolium tetrafluoroborate [EOOEMIM][BF_4_])-modified hollow glass microspheres. 1-aminoethyl-3-methylimidazolium hexafluorphosphate ([APMIM]PF_6_) and [EMIM]PF_6_ were also used as flame-retardant additives for PU.

### 3.7. Carbon Dioxide Capture

Carbon dioxide (CO_2_) is a greenhouse gas, and large amounts of CO_2_ emissions are an important cause of the greenhouse effect and global warming. Therefore, the absorption and dissolution of CO_2_ are particularly important [141,142]. It has been reported that IL has excellent characteristics of the high solubility of CO_2_ [143]; however, due to the high viscosity and high cost of IL [144], its application in CO_2_ capture is limited. By combining IL and polymers [145], many researchers not only solved the problem of IL in CO_2_ capture but also improved the efficiency of CO_2_ absorption.

Jourdain et al. [97] synthesized a cationic PU using a 1, 2, 3-triazolium-functionalized diol monomer containing a [TFSI]^−^ counter-anion and explained the influence of PU structure, porosity, and specific surface area of the film on the effect of CO_2_ absorption. Fernandez et al. [102] synthesized a PU foam via the polyaddition of castor oil and hexamethylene diisocyanate (HDI), which was mixed with IL ([BMIM]Cl, [BMIM]BF_4_, or [EOHMIM]Gly). It was found that the content of IL would affect the pore size and density of PU foam and influence CO_2_ absorption further. It was found that the foam containing 15 wt% [BMIM]Cl showed a higher trapping capacity than the foam without IL. The influence of anion and cation structures of IL on the absorption of CO_2_ has also been studied. Luz et al. [103] prepared two different blends of cationic IL and PU, and the results showed that imidazolium cation was more conducive to the absorption of CO_2_ than phosphonium cation. Morozova et al. [146] prepared ionic PUs using four different ionic diols with thirteen different counterions and studied the effect of diisocyanate monomers and cation/anion structures on the CO_2_-capturing ability. It was concluded that the anion variation was the key controlling factor affecting the absorption of CO_2_ by ionic PU. The most significant advantage of the synthesized ionic PU was that it achieved the highest CO_2_ absorption capacity (up to 24.76 mg g^−1^ at 1 bar and 273 K) known so far for poly(ionic liquid)s.

### 3.8. Biomedical Applications

PU is an attractive polymer for biomedical applications due to its compatibility with biological tissue and designable diversity in structure and properties [147]. For example, shape memory PU is an attractive member in health rehabilitation because of its malleable performance at temperatures higher than *T*_g_ or *T*_m_ of the soft segment phase. By cooling or heating PU, the cyclic shape memory effect can be achieved. In addition, for biomedical materials, anti-bacterial activity should be taken into consideration. Meng et al. [148] synthesized a cationic shape memory PU using N, N-bis(2-hydroxyethyl)isonicotinamide as a chain extender, which was neutralized with acetic acid. The cationic PU showed high mechanical properties, good biocompatibility, non-toxicity, and antibacterial properties. It was an excellent candidate for low-temperature thermoplastic antibacterial orthotic materials. To avoid the medical catheter-related infection, Xiu et al. [105] synthesized a novel imidazolium IL, Bim-BU, as a cationic antibacterial agent to modify PU via the polymer blending technique. The prepared PU/Mim-BU showed highly efficient and long-term antibacterial abilities against Gram-positive and Gram-negative bacteria. Its good biocompatibility was also proven through cytotoxicity and hemolysis experiments. To improve the blood compatibility of PU, cationic PU was prepared by introducing carboxylic groups into the segments of PU chains by means of using dimethylolpropionate as a chain extender [149,150] or grafting thioglycolic acid into the soft segment. It was found that the insertion of the carboxylic groups on the soft segments would promote blood compatibility and reduce the formation of blood clots [104].

In addition to the above-mentioned applications, IL-modified PUs have also been used in thermochromic sensors [79], shape memory materials [151], electromagnetic interference shielding [152], and many other fields.

## 4. Conclusions

PU has excellent wear resistance, structural adjustable, and processable properties and thus can be widely applied as coating materials, elastomers, soft and hard foam plastics, and artificial leather, involving the fields of textiles, medicine, food, construction, etc. After introducing ILs into the PU matrix, PU can be endowed with conductivity, flame retardancy, antibacterial properties, etc., making it suitable for strain/pressure sensors, actuators, transistors, antistatic, and flame-retardant applications. In this review, we summarized the fabrication methods for IL-modified PU, including the simple physical blending method by solvent casting and a relatively complicated chemical method by copolymerization. By physical blending, a large amount of ILs can be added to the PU matrix, but IL leakage sometimes occurs, which reduces the long-term stability of the PU-IL system. It can be avoided by improving the physical interaction between ILs and the PU matrix, for example, hydrogen bond interaction. By the copolymerization method, ILs are covalently introduced into PU as parts of the backbone or end-capped groups. The performance of IL-modified PU can be widely adjusted by the chemical structures of the ILs and PU polymer chains, which significantly influence the interaction between ILs and the PU matrix and further determine the application of the ionic PU. Then, we summarized the applications of IL-modified PU, including sensors, actuators, transistors, antistatic films, solid-state electrolytes, flame-retardant elastomers, carbon dioxide capture, and biomedical applications. It is noted that IL-modified PU has an extremely wide range of applications. In addition, due to the structural diversity of ILs and PU, there are more possibilities for the structural design of ionic PU as well as more applications. However, there are still many issues, such as its poor conductivity, low stability, inability to permanently antistatic, and easy leakage of ILs, that need to be solved not only considering the performance but also analyzing the synthesis route of ionic PU to balance the effectiveness and simplicity of solutions. In the future, related studies should focus on the interaction between ionic groups and PU to design and prepare a new generation of ionic PU while solving the present problems and improving the performance of ionic PU to meet the more complicated requirements of new applications.

## Data Availability

Not applicable.
